# Aryl hydrocarbon receptor and IL-13 signaling crosstalk in human keratinocytes and atopic dermatitis

**DOI:** 10.3389/falgy.2024.1323405

**Published:** 2024-01-26

**Authors:** Steven P. Proper, Alexander T. Dwyer, Andrews Appiagyei, Jennifer M. Felton, Netali Ben-Baruch Morgenstern, Justin M. Marlman, Michael Kotliar, Artem Barski, Ty D. Troutman, Marc E. Rothenberg, Tesfaye B. Mersha, Nurit P. Azouz

**Affiliations:** ^1^ Division of Allergy and Immunology, Cincinnati Children's Hospital Medical Center, Cincinnati, OH, United States; ^2^ Division of Human Genetics, Cincinnati Children's Hospital Medical Center, Cincinnati, OH, United States; ^3^ Department of Pediatrics, University of Cincinnati College of Medicine, Cincinnati, OH, United States; ^4^ Division of Asthma Research, Cincinnati Children's Hospital Medical Center, Cincinnati, OH, United States

**Keywords:** aryl hydrocarbon receptor, keratinocytes, atopic dermatitis, IL-13, STAT6, tapinarof, FICZ

## Abstract

**Introduction:**

Atopic dermatitis (AD) is an allergic skin disease mediated by skin barrier impairment and IL-13-driven immune response. Activation of the aryl hydrocarbon receptor (AHR) has shown promise in early clinical trials for AD; however, the mechanism by which AHR partially ameliorates AD is not well known.

**Methods:**

Gene expression data from human biopsies were analyzed, and compared to gene expression from RNA-sequencing in our *in-vitro* HaCaT cell model system. Western blot, ELISA qRT-PCR were used to further explore the relationship between AHR and IL-13 signaling in HaCaT cells.

**Results:**

The AHR target gene *CYP1A1 was* decreased in lesional skin compared with healthy control skin (*p* = 4.30 × 10^−9^). Single-cell RNA sequencing (scRNAseq) demonstrated increased *AHR* expression (*p* < 1.0 × 10^−4^) and decreased *CYP1A1* expression in lesional AD keratinocytes compared with healthy control keratinocytes (*p* < 0.001). Activation of AHR by AHR agonists in HaCaT cells reversed IL-13-dependent gene expression of several key genes in AD pathogenesis, most notably the eosinophil chemoattractant *CCL26* (eotaxin-3). Differentially expressed genes in keratinocytes of patients with AD substantially overlapped with genes regulated by AHR agonists from HaCaT cells by RNAseq, but in reverse direction. Mechanistically, there was evidence for direct transcriptional effects of AHR; AHR binding motifs were identified in the differentially expressed genes from lesional AD keratinocytes compared to control keratinocytes, and AHR activation did not modify IL-13-dependent signal transducer and activator of transcription 6 (STAT6) translocation to the nucleus.

**Discussion:**

Together, these data suggest that the AHR pathway is dysregulated in AD and that AHR modulates IL-13 downstream signaling in keratinocytes through genome-wide, transcriptional regulatory effects.

## Introduction

Atopic dermatitis (AD) is a chronic, relapsing inflammatory skin disorder with a hallmark of impaired skin barrier function affecting up to 11% of children and 7% of adults in the US and up to 20% of people worldwide and constantly increasing in prevalance (1). AD is considered to be a major risk factor for the development of other allergic diseases (2). A combination of environmental and genetic factors contributes to the local and systemic inflammation that drives AD and other atopic conditions. Given the importance of environmental factors in AD specifically, significant attention has been focused in recent years on the aryl hydrocarbon receptor (AHR), an environmental sensor and canonical member of the Per-Arnt-Sim (PAS) superfamily of proteins, and its role in the skin (3).

AHR was originally identified due to its role in mediating the toxicity of xenobiotics [e.g., 2,3,7,8-tetrachlorodibenzo-p-dioxin (TCDD)]. Although classical synthetic ligands of AHR produced toxic effects [including the clinical skin finding “chloracne”, a cystic inflammatory condition caused by exposure to polychlorinated biphenyls (PCBs) and similar compounds], significant efforts were made to identify the natural ligands and functions of AHR. This work revealed several ligands that are likely important for the skin and AD. One such endogenous ligand is 6-formyl-indolo[3,2-b]carbazole (FICZ), a product of tryptophan, which is abundant in the skin, created by UV radiation or microbial metabolism (4). UV radiation being an effective treatment strategy for AD has driven further interest in AHR activation as a potential therapeutic mechanism (5). Indeed, a growing body of research has demonstrated that AHR activation with non-TCDD ligands can improve skin barrier function, presumably through epidermal differentiation complex genes and others (6–8) .

Other AHR-activating ligands with possible relevance for the skin could be tryptophan metabolites such as kynurenine (9), indoles [created by commensal microbiota (10)], particulate matter, and other products of combustion (11). Notably, AHR activation with microbiome-derived ligands did not reproduce the toxic effects seen with dioxins (e.g, TCDD) and other toxicants, which is thought to be related to the half-life of these compounds and intensity of AHR activation (12). However, further studies are needed to understand the complex interplay of AHR ligands in AD. Tapinarof, a naturally occurring (now fully synthetic) AHR agonist, has shown promise in safely treating AD through phase 2 clinical trials, with phase 3 clinical trials underway (8, 13, 14). The mechanism by which Tapinarof improves AD is not fully understood. Unanswered questions include the primary target cell type (keratinocytes vs. immune cells) and the underlying signaling responsible for these changes (improvement of skin barrier function vs. disruption of IL-13 signaling).

Keratinocytes are the primary functional cells of the epidermis and form an interactive, physical barrier that directs innate immune responses. As the first line of defense from the outside environment, keratinocytes are uniquely positioned to have significant exposure to environmental AHR ligands. Given this unique role of keratinocytes, we hypothesized that AHR signaling in keratinocytes has an active role in AD. To gain more insight into the role of AHR in these cells and the broader implications for AD, we sought to investigate how AHR activation affects IL-13 signaling in keratinocytes both from patients with AD and *in vitro* models of AD.

Herein, we provide evidence that AHR signaling is dysregulated in keratinocytes from patients with AD on the basis of gene expression data. We found that AHR activation blocked IL-13–dependent expression of key genes in AD pathogenesis, including *CCL26* (eotaxin-3), a crucial cytokine gene regulated by STAT6. We showed substantial overlap of the AHR-regulated genes and the AD transcriptome genes in keratinocytes; however, the expression of these overlapping genes changed in opposing directions (upregulated vs. downregulated genes) in AHR-regulated vs. AD transcriptome contexts. Analyzing STAT6 did not reveal substantial AHR-regulated changes in STAT6 expression, phosphorylation, nor nuclear translocation. In contrast, we demonstrated that among open chromatin regions from keratinocytes in AD lesional skin, AHR binding motifs were enriched near the transcriptional start site of differentially regulated genes. These data suggest that DNA binding is the primary mechanism by which AHR activation alters type 2 signaling in keratinocytes and highlight the need to further elucidate these molecular mechanisms due to AHR activation being utilized to treat AD.

## Methods

### Cell culture and reagents

HaCaT cells were obtained from CLS (item no. 300493, Eppelheim, Germany) and validated via STR profiling by Labcorp (Burlington, North Carolina, USA). HaCaT cells were cultured in Gibco DMEM from Thermo Fischer (Cat. No. 10567014, Waltham, MA, USA), supplemented with 10% Fetal Bovine Serum (FBS) from R&D Biosystems (Cat. No. S11150, Minneapolis, MN, USA) and 1% Penicillin/Streptomycin from Gibco/Thermo Fisher Scientific (Cat. No. 15140-122, Waltham, MA, USA). Cells were plated onto Falcon brand cell culture plates from Corning (Corning, NY, USA) and kept at 37°C and 5% CO_2_. Passaging of cells was carried out with 0.05% trypsin from Gibco/Thermo Fisher Scientific (Cat. No. 25300-054, Waltham, MA, USA) and 1× phosphate-buffered saline that was diluted from 10× phosphate-buffered saline from Gibco/Thermo Fisher Scientific (Cat. No. 14200-075, Waltham, MA, USA) with purified water from a NanoPure filtration system (Thomas Scientific, Swedesboro, NJ, USA) and then sterilized by autoclave. HaCaT cells were grown to confluence and then cultured for an additional 3 days to ensure that a monolayer was achieved prior to dosing. Dimethylsulfoxide (DMSO) was obtained from Sigma-Aldrich (Cat. No. D2650, St. Louis, MO, USA) and used to dilute FICZ, tapinarof, and GNF351 prior to their final dilution in culture media. FICZ was obtained from Tocris/Bio-Techne (Cat. No. 5304, Minneapolis, MN, USA), put into 1 mM stock solution with DMSO kept at −20°C, and protected from light prior to use in experiments, for which it was further diluted into culture media at 1:1,000 to a final concentration of 1 µM. Tapinarof was obtained from MedChemExpress (Cat. No. HY-109044, Monmouth Junction, NJ, USA), put into 1 mM stock solution with DMSO kept at −20°C, and similarly diluted with culture media to a final concentration of 1 µM. GNF351 was obtained from EMD Millipore (Cat. No. 182707-10MG, Burlington, MA, USA), put into 1 mM stock solution with DMSO kept at −20°C, and diluted with culture media to a final concentration of 1 µM. Unless otherwise noted, chemical reagents were obtained from Sigma-Aldrich (St. Louis, MO, USA). All treatment reagents were added simultaneously without pre-incubation of any reagents prior to extraction. After dosing, cells were washed with 1× ice-cold PBS prior to isolation of either protein or RNA as described below.

### mRNA extraction and quantitative RT-PCR

Total RNA was isolated from cells using TriPure Isolation Reagent from Sigma-Aldrich using the manufacturer's instructions (Cat. No. 11667165001, St. Louis, MO, USA). The RNA layer was further purified using the RNeasy Mini Kit by Qiagen (Cat. No. 2170004, Germantown, MD, USA) according to the manufacturer's instructions. cDNA was created from RNA using Protoscript First Strand cDNA Synthesis Kit from New England BioLabs (Cat. No. E63005, Ipswitch, MA, USA). qPCR was performed using an ABI QuantStudio 7 Flex (Thermo Fisher, Waltham, MA, USA) with PowerUp SYBR Green Master Mix from Thermo Fisher (Cat. No. A25742, Waltham, MA, USA) using the following primer sets: *GAPDH* (forward 5′-AGGTCGGAGTCAACGGATTT, reverse 5′- GACGGTGCCATGGAATTTGC), *CYP1A1* (forward 5′-AGTGATTGGCAGGTCACGG, reverse 5′-GTCTCTTGTTGTGCTGTGGGG), and *CCL26* (forward 5′-TCCCAGCGGGCTGTGATATTC, reverse 5′-TCCAAGCGTCCTCGGATGAA).

### Protein extraction and western blot

The nuclear and cytosolic protein fractions from cell cultures were extracted with NE-PER Nuclear and Cytoplasmic Extraction Reagents from Thermo Fisher (Cat. No. 78835, Waltham, MA, USA), and protein from the whole cell fraction and supernatants were extracted with M-PER buffer from Thermo Fisher Scientific (Cat. No. 78501, Waltham, MA, USA) with protease inhibitors from Roche/Sigma-Aldrich (Cat. No. 11836153001, St. Louis, MO, USA). 4× Bolt LDS Sample Loading Buffer from Invitrogen/Thermo Fisher (Cat. No. B0008, Waltham, MA, USA) was added, and samples were sonicated at 10 kHz for two 10-s intervals with a 5-s break between intervals (Fisher Scientific UltraSonic Processor). Samples were then heated to 95°C for 5 min, and cellular debris was spun down at 12,000 g for 5 min before being subjected to electrophoresis on Bolt 4%–12% Bis-Tris gels from Invitrogen/Thermo Fisher (Cat. No. NW04127BOX, Waltham, MA, USA) at 200 V for 30 min, transferred to SureLock Tandem Midi Pre-cut nitrocellulose membranes from Invitrogen/Thermo Fisher (Cat. No. 11836153001, Waltham, MA, USA) at 30 V for 1 h, and visualized using the Odyssey CLx system (LI-COR Biosciences). Membranes were blocked with Intercept TBS Blocking Buffer from Li-Cor (Cat. No. 927-60001, Lincoln, NE, USA) prior to incubation with primary antibodies. Primary antibodies were rabbit anti-AHR monoclonal IgG (Cell Signaling 83200, clone D5S6H, 1:2,000), rabbit anti-pSTAT6 (Tyr641) monoclonal IgG (Cell Signaling 565545, clone D8S9Y, 1:2,000), rabbit anti-Lamin B1 polyclonal IgG (Proteintech 12987-1-AP, 1:2,000), and mouse anti-GAPDH monoclonal IgG (Origene TA802519, clone OTI2D9, 1:2,000). Secondary antibodies were donkey anti-rabbit IgG (Alexa Fluor 790, Jackson ImmunoResearch 711-655-152) or donkey anti-mouse IgG (Alexa Fluor 680, Jackson ImmunoResearch 715-625-150), all a 1:10,000 dilution from a 1.5 mg/ml stock. Blots were quantified using Image J Software (15).

### Cytokine protein analysis

Supernatants from HaCaT cells were collected and centrifuged at 4°C for 5 min at 5,000 g; the middle layers were collected and stored at −80°C until analysis using the Human CCL26/Eotaxin-3 DuoSet ELISA from R&D Systems/Bio-Techne (Cat. No. DY346, Minneapolis, MN, USA).

### Publicly available RNA sequencing data analysis

**Figure 1:**
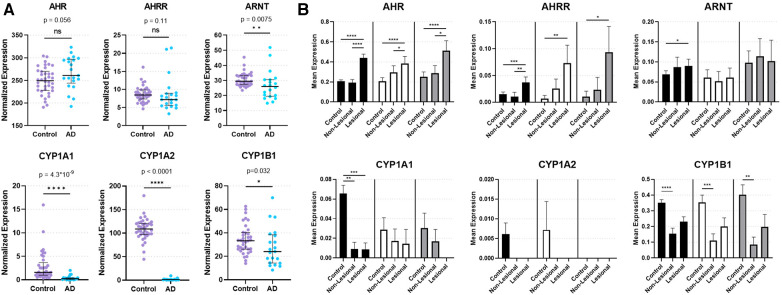
Expression of AHR and key AHR targets genes from skin biopsies. (**A**) Bulk RNA sequencing from control and lesional AD skin biopsies in GSE121212: 38 control subjects and 21 with AD. Median with interquartile range is shown with markers representing individual subjects. Mann–Whitney Test (two-tailed, *α *= 0.05). ns, not significant. *p < 0.05, **p < 0.01, ****p < 0.0001. (**B**) Single-cell RNA sequencing of keratinocytes [KC1 = black bars, corresponding most with basal keratinocytes; KC2 = white bars, corresponding most with suprabasal keratinocytes; KC3 = gray bars, corresponding most with proliferating keratinocytes (17)] from healthy control, non-lesional AD and lesional AD skin biopsies in GSE147424. Bars show mean normalized expression with error bars showing standard error of the mean. Kruskal Wallis test with Dunn's test for multiple comparisons was used. *p < 0.05, **p < 0.01, ***p < 0.001, ****p < 0.0001.

Gene Expression Omnibus (GEO) data accessed for this study from publicly available datasets are as follows: GSE121212 and GSE147424. GSE121212 provided bulk RNA sequencing (RNAseq) from skin biopsies from patients with AD and healthy controls (16), which was plotted as relative expression between control and lesional skin samples for genes of interest. GSE147424 provided single-cell RNA sequencing (scRNAseq) gene expression, and higher order clustering of single cells identified in the original manuscript by He et al. 2020 (17). Keratinocytes from each cluster were identified using the marker genes defined in the manuscript, with KC1 representing basal keratinocytes (high KRT5 and KRT15 expression), KC2 representing suprabasal keratinocytes (high KRT1 and KRT5 expression) and KC3 representing proliferating keratinocytes (high TOP2A and UBE2C expression). Gene expression of AHR and other genes of interest among KC1-KC3 clusters were evaluated in Figure 1. In addition, He et al. provided additional differential gene expression data in Table E3 which included a group called “Keratinocytes” and includes individual cells from all keratinocyte clusters (essentially a combination of KC1, KC2, and KC3). Differentially expressed genes (DEGs) between lesional AD and control keratinocytes (all keratinocytes, clusters KC1 through KC3 combined) were identified as the “AD transcriptome” for keratinocytes. In addition, transcription factor binding motif enrichment analyses of DEGs in lesional AD compared with control keratinocytes (all keratinocytes) from GSE147424 were evaluated using the HOMER software package (18). Briefly, HOMER uses a library of >7,000 transcription factor binding models (in the form of position weight matrices) to scan a set of input sequencing for statistical enrichment of each position weight matrix. Calculations were performed using ZOOPS (zero or one occurrence per sequence) scoring coupled with hypergeometric enrichment analysis to determine motif enrichment. Input enhancer sequences were also assessed for statistical enrichment of motifs for AHR binding sites using the findPeaks program and factor mode within HOMER. Significantly enriched transcription factor binding site motifs are expressed as log *p* values.

### RNA sequencing and analysis

RNAseq libraries were generated as previously described (19, 20) using 500 ng of purified RNA using the Zymo Research Direct-zol RNA microprep kit (Cat. No. R2062, Irvine, CA, USA). In brief, mRNAs were enriched by incubation with Oligo d(T) Magnetic Beads (New England Biolabs, Cat. No. S1419S, Ipswich, MA, USA) and then fragmented/eluted by incubation at 94°C for 9 min. Poly A–enriched mRNA was fragmented in 2× Superscript III first-strand buffer (Invitrogen/Thermo Fisher, Cat. No. 12574026, Waltham, MA, USA) with 10 mM DTT (Thermo Scientific, Cat. No. R0861, Waltham, MA, USA) by incubation at 94°C for 9 min and then immediately chilled on ice before the next step. The 10 µl of fragmented mRNA, 0.5 µl of random primer (Invitrogen/Thermo Fisher, Cat. No. 48190011, Waltham, MA, USA), 0.5 µl of Oligo dT primer (Invitrogen/Thermo Fisher, Cat. No. 18418012, Waltham, MA, USA), 0.5 µl of SUPERase-In (Ambion/Thermo Fisher, Cat. No. AM2694, Waltham, MA, USA), 1 µl of dNTPs (10 mM, Thermo Scientific, Cat. No. R0194, Waltham, MA, USA), and 1 µl of DTT were heated at 50°C for 3 min. At the end of the incubation, 5.8 µl of water, 1 µl of DTT (100 mM), 0.1 µl Actinomycin D (2 mg/ml, Invitrogen/Thermo Fisher, Cat. No. A7592, Waltham, MA, USA), 0.2 µl of 1% Tween-20 (Sigma-Aldrich, Cat. No. P1379, St. Louis, MO, USA), and 0.2 µl of Superscript III (Invitrogen/Thermo Fisher, Cat. No. 18080093, Waltham, MA, USA) were added and incubated in a PCR machine using the following conditions: 25°C for 10 min, 50°C for 50 min, and a 4°C hold. The product was then purified with RNAClean XP beads (Beckman Coulter, Cat. No. A63987, Indianapolis, IN, USA) according to the manufacturer's instructions and eluted with 10 µl nuclease-free water. The RNA/cDNA double-stranded hybrid was then added to 1.5 µl of Blue Buffer (Enzymatics, Cat. No. B0110, Beverly, MA, USA), 1.1 µl of dUTP mix (10 mM dATP, dCTP, dGTP and 20 mM dUTP, Enzymatics, Cat. No. N2050-10-L, Beverly, MA, USA), 0.2 µl of RNase H (5 U/ml, Enzymatics, Cat. No. Y9220l, Beverly, MA, USA), 1.05 µl of water, 1 µl of DNA polymerase I (Enzymatics, Cat. No. P7050l, Beverly, MA, USA), and 0.15 µl of 1% Tween-20. The mixture was incubated at 16°C for 1 h. The resulting dUTP-marked double-stranded DNA was purified using 28 µl of Sera-Mag Speedbeads (Cytiva, Cat. No. 65152105050250, Marlborough, MA, USA), diluted with 20% PEG8000 (2.5M NaCl) to a final of 13% PEG8000, eluted with 40 µl EB buffer (10 mM Tris-Cl, pH 8.5), and frozen at −80°C. The purified double-stranded DNA (40 µl) underwent end repair by blunting, A-tailing, and adaptor ligation as previously described (18) using indexed barcoding adapters (Perkin Elmer, NEXFLEX Unique Dual Indexing Barcodes). Libraries were PCR-amplified for 9–14 cycles, purified with Sera-Mag Speedbeads, and quantified using a Qubit dsDNA HS Assay Kit (Thermo Fisher Scientific, Cat. No. Q32854, Waltham, MA, USA). RNAseq libraries were sequenced using PE150 and an SP type flow cell on a NOVASeq 6000 at the Cincinnati Children's Hospital Medical Center DNA Sequencing and Genotyping Core Facility. Sequencing data were mapped with STARR (21) to the GRCh38 reference genome, and sequencing counts were generated using HOMER analyzeRepeats.pl (18). Differentially expressed genes (DEGs) were identified with DeSeq2 (22) using the HOMER wrapper script, getDiffExpression.pl. We filtered genes to those with a minimum transcript per million (TPM) value >8 (to remove low-transcript level genes), absolute fold change >1.5, and adjusted *p*-value < 0.05 (when comparing any 2 of the 3 groups) and performed hierarchical clustering with Morpheus (Broad Institute) using a “one minus Pearson correlation” and “average linkage” method. Gene ontology enrichment analysis, which uses statistical methods to determine functional pathways and cellular processes associated with a given set of genes, was performed with the ToppGene suite (Cincinnati Children's Hospital Medical Center, https://toppgene.cchmc.org/navigation/termsofuse.jsp) (23). Principal component analysis (PCA) from gene expression data was performed using GraphPad Prism 9 (GraphPad Software, San Diego, CA, USA) with the standard method, and principal components were selected using parallel analysis with eigenvalues greater than those from 1,000 simulations at the 95th percentile with auto random seed.

### Statistical analyses

Statistical analysis of publicly available gene expression data, quantitative reverse-transcriptase PCR gene expression of HaCaT cells, and cytokine protein quantification with ELISA was performed and graphed using GraphPad Prism 9 (GraphPad Software, San Diego, CA, USA).

## Results

### AHR signaling in patients with AD

In order to investigate the natural state of AHR expression and genes involved in regulation of AHR-mediated downstream events in AD, we analyzed bulk RNAseq data (GSE121212) of skin biopsies from 21 subjects with AD (lesional skin) and 38 control subjects (healthy skin). *AHR* expression trended toward an increase in lesional skin compared to control skin (*p* = 0.056; Figure 1A). In contrast, the AHR target gene *CYP1A1* was significantly decreased in lesional skin (*p* = 4.30 × 10^−9^; Figure 1A), as was *CYP1A2* (*p* < 0.0001; Figure 1A) and *CYP1B1* (*p* = 0.032; Figure 1A). Expression of the AHR repressor (*AHRR*) was not statistically different between healthy control and lesional skin (*p* = 0.11; Figure 1A), though the Aryl hydrocarbon Receptor Nuclear Translocator (*ARNT)* expression was decreased in lesional skin compared to controls (*p* = 0.0075; Figure 1A). Focusing further on keratinocytes, we analyzed publicly available single-cell RNA sequencing (scRNAseq) data from biopsies of subjects with AD compared to healthy controls (Figure 1B). We analyzed the three major keratinocyte populations [designated KC1, KC2 and KC3 in He et al. 2020 (17); GSE147424] with scRNAseq data of 5 AD subjects (from lesional and non-lesional skin biopsies) and 6 healthy control subjects (healthy skin biopsies). *AHR* expression was significantly increased in lesional keratinocytes compared to healthy control (*p* < 0.0001 in KC1, KC2, and KC3) and non-lesional keratinocytes (*p* < 0.0001 for KC1, *p* < 0.05 for KC2 and KC3, respectively; Figure 1B). In contrast, *CYP1A1* expression was significantly decreased in both lesional and non-lesional keratinocytes compared to healthy controls (*p* < 0.001 and *p* < 0.01, respectively; Figure 1B), though this was only evident in the KC1 population. *CYP1A2* expression was not reliably detected in these keratinocyte populations, suggesting the source of *CYP1A2* expression in bulk RNA sequencing samples (Figure 1A) may not have been keratinocytes. *CYP1B1* expression was decreased in non-lesional keratinocytes compared with control keratinocytes (*p* < 0.0001 for KC1, *p* < 0.001 for KC2, and *p* < 0.01 for KC3; Figure 1B) *ARNT* expression was higher in lesional keratinocytes compared to healthy controls only for KC1 (*p* < 0.05; Figure 1B), and was not different across KC2 or KC3 populations. Interestingly, *AHRR* expression was increased in lesional keratinocytes compare to controls (*p* < 0.001 in KC1, *p* < 0.01 in KC2, and *p* < 0.05 in KC3; Figure 1B) and only in KC1 was *AHRR* expression higher in lesional than non-lesional keratinocytes (Figure 1B). These data suggest that despite increased *AHR* expression, lesional keratinocytes do not demonstrate canonical *CYP1A1, CYP1A2* or *CYP1B1* activation. The attenuated expression of AHR target genes may stem from the increased expression of *AHRR* in these lesional keratinocytes from patients with AD at least in part. In addition, the decreased *ARNT* expression seen in bulk RNA samples may also contribute to the decrease in *CYP1A1, CYP1A2* and *CYP1B1* expression in AD samples (Figure 1A). Overall these data imply that the AHR signaling pathway may be dysregulated in keratinocytes of AD patients.

### AHR activation by FICZ in HaCaT keratinocyte model

**Figure 2:**
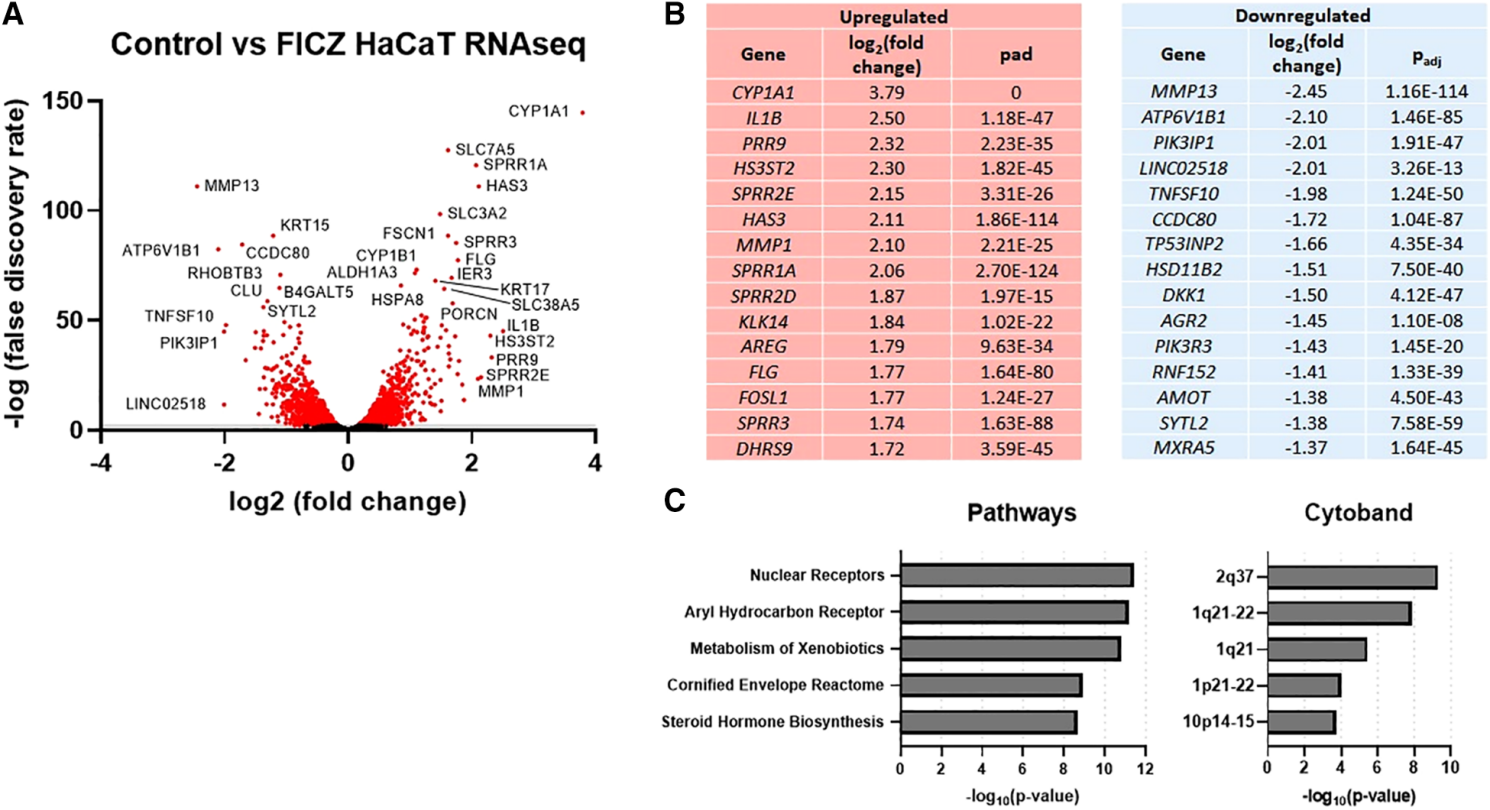
RNAseq gene expression of control vs. FICZ-treated HaCAT cells. (**A**) Volcano plot showing log_2_, (false discovery rate) of all genes detected by RNAseq (6,979 genes), with red indicating all genes with –log_10_ (false discovery rate) <2 (2,866 genes), with many of the most significant genes labeled. (**B**) Top 15 upregulated and downregulated genes are listed with log_2_ (fold change) and adjusted *p*-value (*P*_adj_). (**C**) Top 5 pathways and cytobands are shown from gene ontology analysis of all genes with absolute fold change >1.5 (725 genes) using ToppFun.

In order to further explore gene expression regulated by AHR in keratinocytes, we stimulated HaCaT cells, a human keratinocyte cell line (24), with FICZ. RNAseq was used to measure gene expression, as summarized in Figure 2. The volcano plot of all genes is shown in Figure 2A, and the genes with the most increased and decreased expression are shown in Figure 2B. *CYP1A1*, the canonical AHR target gene, was the most upregulated gene by FICZ stimulation [log_2_ (fold change) of 3.79]. Interestingly, several genes of the epidermal differentiation complex (EDC) on chromosome 1q21, were also upregulated. Notably, EDC genes play an important role in terminal differentiation of the human epidermis and structurally and functionally contribute to skin barrier function (25). These genes include *PRR9* [log_2_ (fold change) of 2.32]; *SPRR2E* [log_2_ (fold change) of 2.15]; *SPRR1A* [log_2_ (fold change) of 2.06]; *SPRR2D* [log_2_ (fold change) of 1.87]; *SPRR3* [log_2_ (fold change) of 1.74], which is reduced in AD skin and inversely correlated with pruritis (itch) in non-lesional AD skin (26); and *FLG* (log_2_ (fold change of 1.77). Notably, mutations in *FLG* are associated with AD (27). The most downregulated gene, *MMP13* [log_2_ (fold change) of −2.45], is known to be downregulated by AHR in the bone (28) and chondrocytes (29). The most significant pathways associated with these DEGs were “nuclear receptors”, “aryl hydrocarbon receptor”, “metabolism of xenobiotics”, “cornified envelope reactome”, and “steroid hormone biosynthesis”. Further, significant cytobands included the 2q37 locus, which contains several UDP glucuronosyltransferase family enzymes (UGT1A family, known targets of AHR 30) and two regions of 1q21 (known cytoband for EDC genes and *ARNT*, a nuclear dimeric partner of AHR, Figure 2C). Overall, these data show that treatment of HaCaT cells with FICZ results in a robust induction of AHR signaling and genes related to the cornified envelope of skin.

### IL-13 treatment in HaCaT keratinocyte model

Previous work has demonstrated the disruptive effects of IL-13 on keratinocytes, differentiation and barrier function (31). To determine the effect of the IL-13 on a HaCaT cell model, we measured gene expression by RNAseq (Supplementary Figure S1). Volcano plot of all genes is shown in Supplementary Figure S1A, and genes with the most increased and decreased expression are shown in Supplementary Figure S1B. The most upregulated gene was *CDH26* [log_2_ (fold change) of 4.50], which is known to be associated with the allergic gastrointestinal diseases eosinophilic gastritis and eosinophilic esophagitis (32). *SERPINB4* and *SERPINB3* were also significantly upregulated [log_2_ (fold change) of 4.09 and 3.21], respectively) and are known to be correlated with AD severity and are increasingly recognized as a biomarker in skin inflammation (33–35) . *PADI3* was upregulated with IL-13 treatment [log_2_ (fold change) of 3.61] and is known to be associated with processing of filaggrin (36). *RPTN*, part of the epidermal differentiation complex, was upregulated with IL-13 treatment [log_2_ (fold change) of 3.35]. *RPTN* is upregulated in AD, and genetic variants in *RPTN* associate with AD severity, early onset of AD, itch, and concomitant asthma (37, 38). *CCL26* (eotaxin-3), a chemokine that is required for eosinophil recruitment (39) and is associated with extrinsic AD (40) and early onset AD in children (41), was upregulated with IL-13 [log_2_ (fold change) of 3.20]. *ANO1,* which is associated with allergy and itch signaling (42, 43), was also upregulated with IL-13 treatment [log_2_ (fold change) of 2.45]. Gene ontology analysis showed that formation of the cornified envelope was the top pathway identified from DEGs and that the 15q15 locus was the most significantly associated cytoband among IL-13–treated HaCaT cells (Supplementary Figure S1C). Of note, 15q15 is a known AD risk locus (44, 45). Together, these data suggest that IL-13 treatment of HaCaT cells drives genes and pathways relevant for AD.

### Overlap between the gene signature of AHR-activated keratinocytes and the AD transcriptome

**Figure 3:**
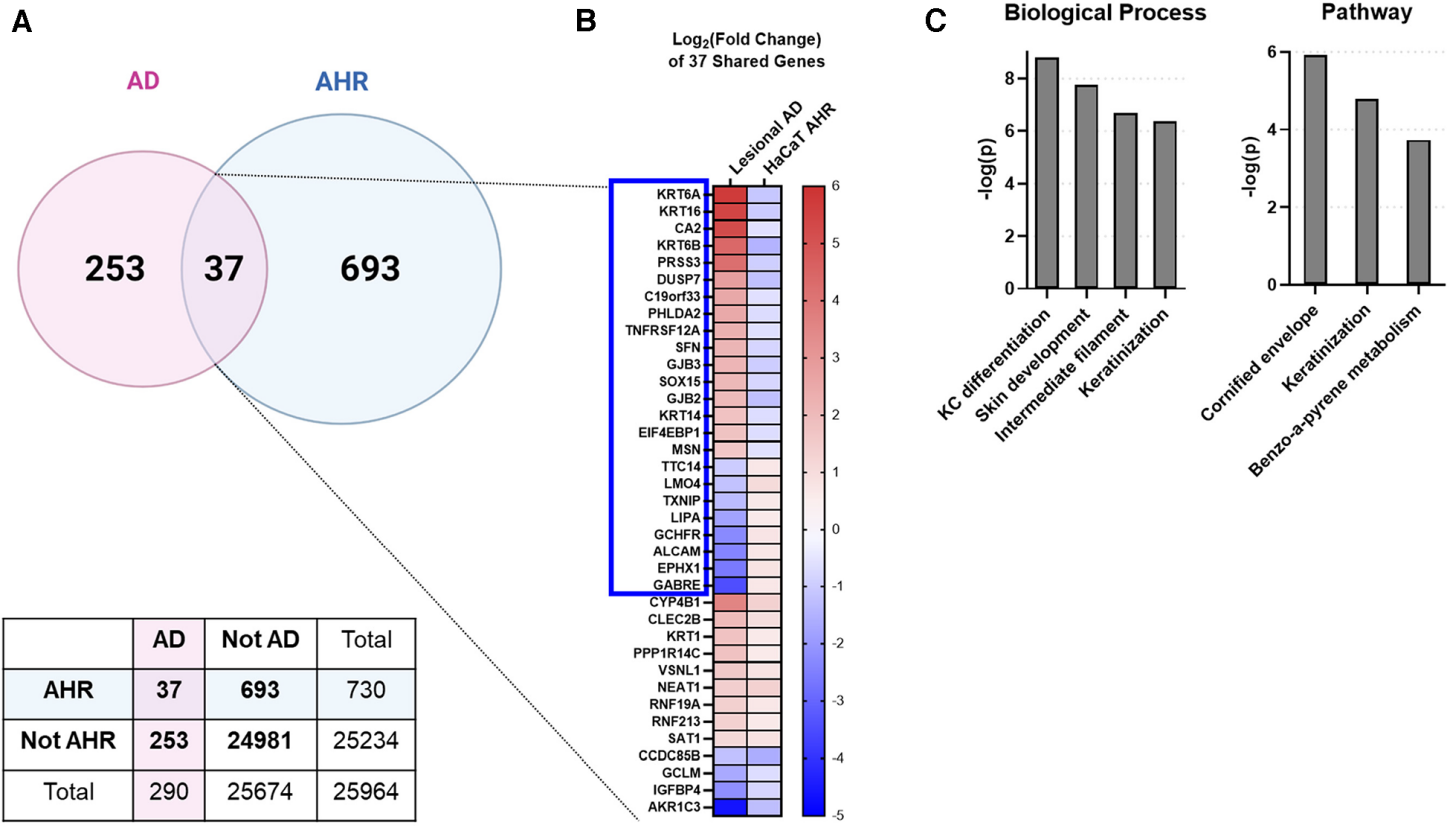
Intersecting the AD transcriptome and AHR transcriptome. We compared the differentially expressed genes (DEGs) from AD lesional keratinocytes (lesional AD vs. control from GSE147424) to HaCaT AHR-regulated genes (FICZ treatment vs. control, RNA sequencing). (**A**) Venn diagram showing unique DEGs from keratinocytes of AD lesional biopsies (253 genes), unique DEGs regulated by AHR (693 genes), and DEGs shared by these two groups (37 genes, 12.8% of AD transcriptome). A contingency table is also shown, and the odds that the 37 shared genes between AD and AHR are by chance is 1.8 × 10^−14^ (Fisher's exact test). (**B**) Heat map of the log_2_ (fold change) of the 37 shared genes in (**A**), sorted first by genes that changed direction (blue box, 24/37 genes, –2/3 of genes), followed by fold change (high to low) in the AD group. (**C**) Gene ontology relationships of the 37 shared genes. KC, keratinocyte. Venn Diagram in (**A**) created with BioRender.com.

To investigate whether an overlap exists between AHR target genes and genes regulated in AD keratinocytes, we compared DEGs from all keratinocytes (clusters KC1 through KC3) of patients with AD [lesional AD vs. healthy controls from GSE147424 and He et al. 2020 (17), referred to here as “AD transcriptome”] with DEGs from our analysis of HaCaT cells treated with an endogenous AHR ligand, FICZ, for 24 h (FICZ vs. control, referred to here as “AHR transcriptome”) (Figure 3). A total of 290 genes were differentially expressed in the AD transcriptome and 730 genes in the AHR transcriptome. Thirty-seven genes overlapped between the AHR transcriptome and the AD transcriptome (12.8% of AD transcriptome; Figure 3A). Further analysis of these 37 overlapping genes using Fisher's exact test revealed that the odds that these 37 genes were shared by chance was 1.8 × 10^−14^ (given the 25,964 genes measured with RNAseq, see contingency table in Figure 3A). When the log_2_ (fold change) of each of these 37 shared genes was compared, 24 of the 37 overlapped genes (approximately 2/3) showed the opposite direction of expression between the AD and AHR activation condition (Blue Box in Figure 3B). Gene ontology analysis of the 37 overlapping genes revealed significant enrichment of the biologic processes of keratinocyte differentiation, skin development, and cornification and in the cellular components of keratin filament, intermediate filament, and connexin complex (Figure 3C). Lesional keratinocytes in AD are less differentiated and express higher levels of markers typically found in the stratum basale layer (46). For example, lesional keratinocytes from the AD transcriptome here demonstrate expected higher expression levels of *KRT6*, *KRT14* and *KRT16* which are reversed by AHR activation with FICZ. These data suggest that AHR activation in keratinocytes affects many shared DEGs involved in keratinocyte differentiation and barrier function.

**Figure 4:**
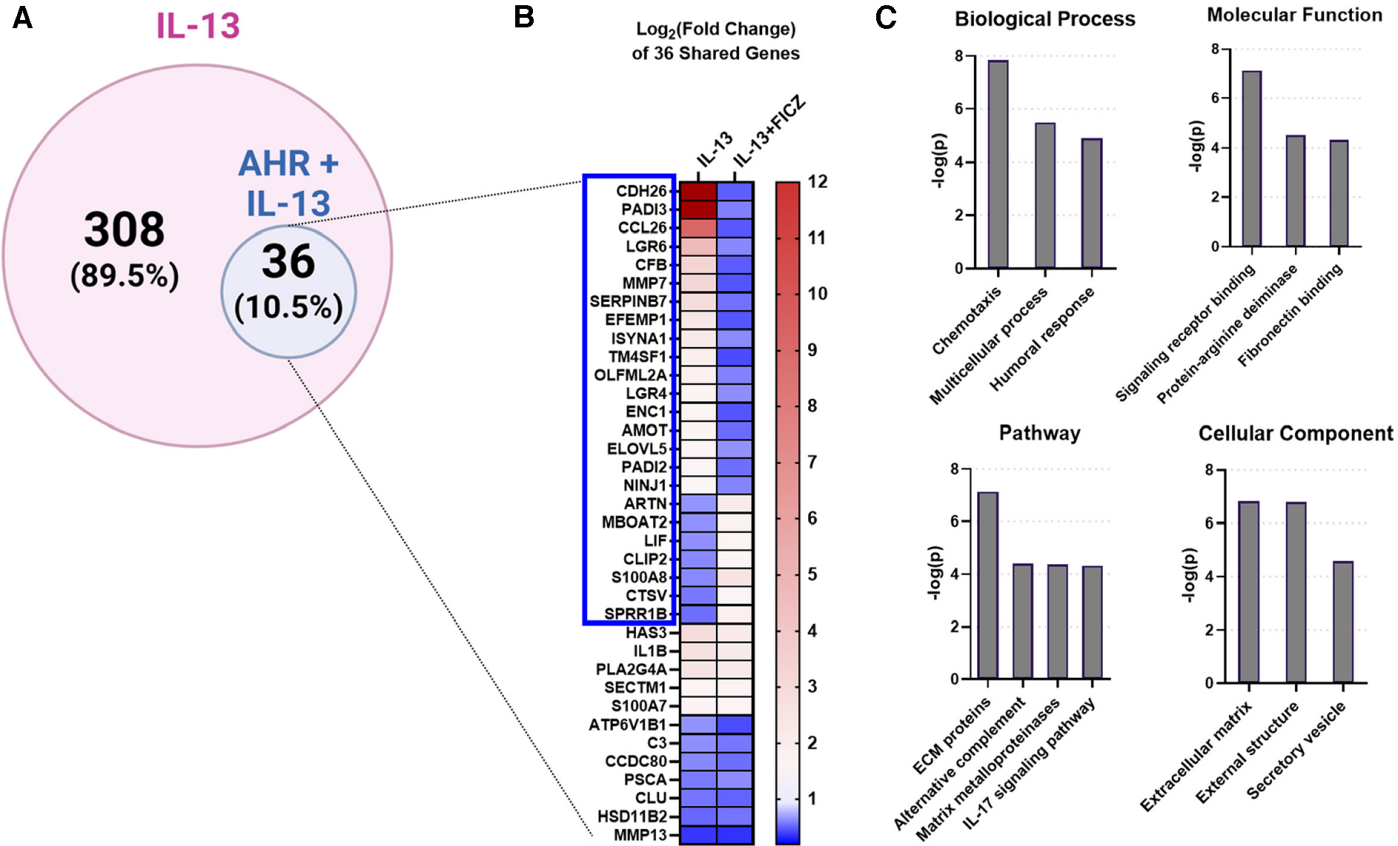
IL-13-regulated genes also regulated by AHR in HaCaTcells. Using RNA sequencing analysis of HaCaT cells, we compared the differentially expressed genes (DEGs) from IL-13 treatment [control vs. IL-13 (10 ng/ml) for 24 h] to DEGs from IL-13 + FICZ treatment) IL-13 [10 ng/ml] vs. IL-13 [10 ng/ml] + FICZ [1 µM]) to model AD vs. AD + AHR activation. (**A**) Diagram showing the 344 DEGs regulated by either IL-13 treatment alone (control vs. IL-13, 308 genes) or also regulated by AHR (IL-13 vs. IL-13 + FICZ, 36 genes, 10.5% of IL-13-regulated genes). (**B**) Heat map of the log2-transformed fold change of the 36 shared genes in (**A**), sorted first by genes that changed direction (blue box, 24/36 genes, 2/3 of genes), followed by fold change (high to low) in the IL-13 group. (**C**) Gene ontology relationships of the 36 shared genes were analyzed. ECM, extracellular matrix. Venn diagram in (**A**) created with BioRender.com.

Next, we investigated whether AHR activation can attenuate the IL-13–mediated responses in our *in vitro* HaCaT cell model system. We stimulated HaCaT cells with IL-13 with and without AHR activation (via FICZ). RNAseq analysis demonstrated that IL-13–regulated genes in HaCaT cells (IL-13 vs. Control) could be altered by additional FICZ-mediated AHR activation (IL-13 vs. IL-13 + FICZ; Figure 4). A total of 36 of the 344 IL-13–regulated genes (10.5% of IL-13 transcriptome) were changed by AHR activation (Figure 4A). Twenty-four of the 36 overlapping genes (2/3 or 67%) had the opposite direction of expression between IL-13 and IL-13 + AHR activation conditions (blue box, Figure 4B). Gene ontology of these 36 overlapping genes revealed enrichment of extracellular matrix genes and inflammatory processes, such as chemotaxis, humoral response, alternative complement, and IL-17 signaling (Figure 4C). Together, these data provide further evidence that AHR activation can partially reverse IL-13–dependent gene expression and that these genes are associated with pathways that are likely relevant for AD.

**Figure 5:**
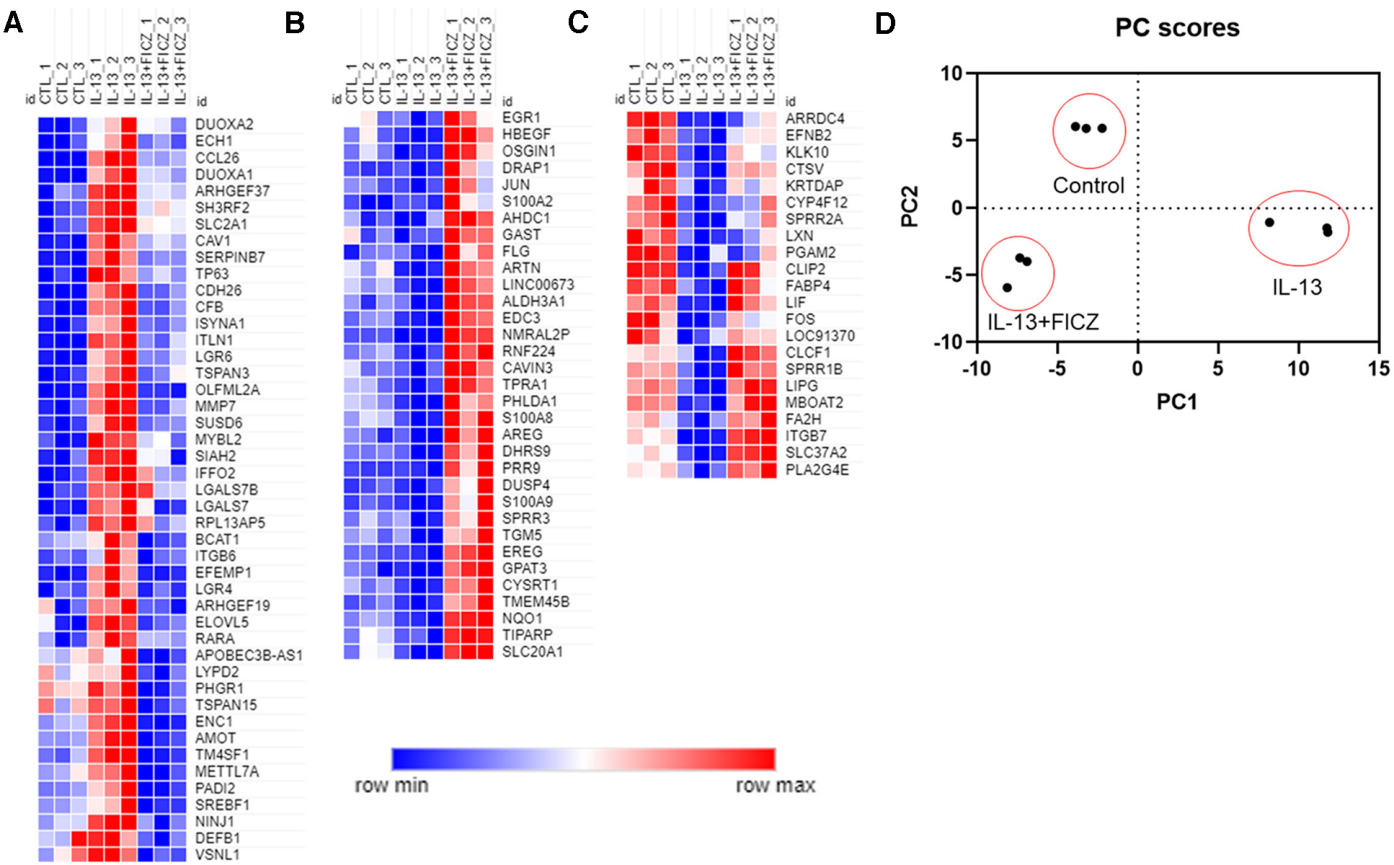
Normalized gene expression patterns of differentially expressed genes in HaCaT cells treated with IL-13 and FICZ. RNA sequencing was performed using confluent HaCaT cells exposed to IL-13 (10 ng/ml) with and without FICZ (1 µM) for 24 h. Genes were limited to those with transcript per million (TPM) >8 and whose absolute fold change was >1.5 and adjusted *p*-value <0.05 (when comparing any 2 of the 3 groups). Normalized gene expression was plotted and colored according to each gene's min and max expression. To better visualize patterns of expression, columns were arranged to show control (CTL), IL-13, and IL-13 + FICZ as follows: (**A**) genes that were upregulated by IL-13 and downregulated by addition of FICZ. (**B**) Low-expression genes that were downregulated by IL-13 and upregulated by addition of FICZ. (**C**) High-expression genes that were downregulated by IL-13 and upregulated by addition of FICZ. (**D**) Principal component analysis (PCA) of gene expression showing first two principal components that clearly discern between each treatment group. Individual markers represent each replicate (*n* = 3 from each treatment group).

We next broadened our analysis of genes in HaCaT cells regulated by both IL-13 and AHR by comparing three groups—control (untreated), IL-13–treated, and IL-13 + FICZ–treated HaCaT cells—and listing any genes that were differentially expressed in any 2 of the 3 comparisons of these groups. This approach is similar to our analysis in Figure 4 except for including DEGs from one additional comparison: control vs. IL-13 + FICZ. Initial hierarchical clustering revealed three distinctive patterns of expression: (1) Genes that were upregulated by IL-13 and downregulated by the addition of FICZ (IL-13 + FICZ group), including the genes *CCL26*, *CDH26*, and *PADI2*, the latter of which is known to differentially regulate Th2/Th17 activation and also play a role in post-translational citrullination required in cornified envelope formation (Figure 5A); (2) Low-expression genes that were downregulated by IL-13 and upregulated by the addition of FICZ, including the genes *FLG*, *SPRR3*, and *ARTN*, all of which are known to be activated by AHR (47, 48) (Figure 5B); and (3) High-expression genes that were downregulated by IL-13 and upregulated by the addition of FICZ, including *FA2H*, a barrier gene contributing to sphingolipid and ceramide synthesis whose downregulation is associated with AD (49), *SPRR1B*, which was downregulated in other cellular models of AD and identified as a “hub” molecule for AD signaling (50), and *CTSV*, which is downregulated in AD and other processes involving desquamation (51) (Figure 5C). Principal component analysis (PCA) confirmed clustering of each treatment group using the first two principal components, supporting uniformity within treatment groups (Figure 5D). We further analyzed the gene ontology of these 100 genes (Supplementary Figure S2). The most significantly related biologic process from these 100 genes was “skin development”, followed by epidermis-related processes, including “plasma membrane organization”, “peptide cross-linking”, “epidermal growth factor receptor (EGFR) activity”, and “epidermal cell differentiation” (Supplementary Figure S2A). The most significant molecular function category was “growth factor activity”, with several other molecular functions related to signaling and fatty acid receptor binding (Supplementary Figure S2B). The most significant pathway identified was “extracellular matrix (ECM)-associated proteins”, and “cornified envelope formation” and “phototherapy-induced nuclear factor erythroid 2-related factor 2 (NRF2)” were notable inclusions (Supplementary Figure S2C). The only two cellular components were “cornified envelope” and “AP-1 complex” (Supplementary Figure S2D). Gene expression and fold change values for these 100 genes are listed in Supplementary Table S1. Together, these findings suggest that IL-13 and AHR share target genes involving epithelial function and that the expression of these genes driven by IL-13 and AHR are generally in the opposite direction.

### Activation of AHR signaling attenuates IL-13: mediated CCL26 expression

**Figure 6:**
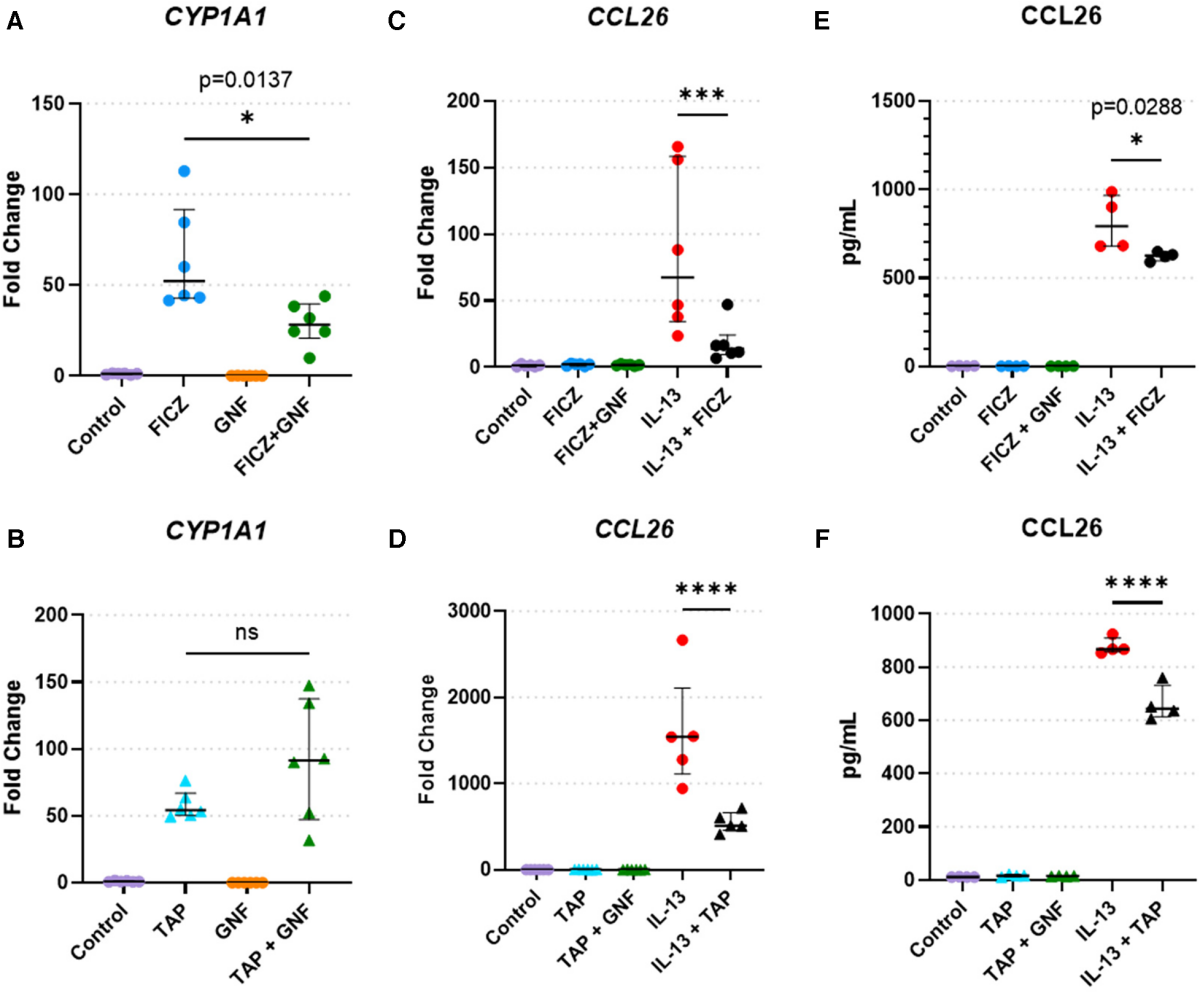
HaCaT cell responses to AHR activation, AHR blockade, and IL-13. (**A**,**B**) CYP1A1 expression in HaCaT cells treated with the (**A**) AHR endogenous ligand FICZ (1 µM) or (**B**) tapinarof (1 µM, TAP), and/or GNF-351 (1 µM, GNF, AHR blocker) (*n* = 6 per group). (**C**,**F**) CCL26 expression in HaCaT cells treated with (**C**). FICZ (1 µM) or (**D**) tapinarof (1 µM) (*n *= 6 per group). (**E**,**F**) CCL26 protein level in HaCaT cells treated with (**E**) FICZ (1 µM) or (**F**) tapinarof (1 µM) (*n* = 4 per group). All treatment reagents were added simultaneously without pre-incubation of any reagents for 24 h prior to extraction. Data are presented as individual replicates with black bars indicating median with interquartile range. One-way ANOVA with Tukey's correction for multiple comparisons. ns, not significant, *p < 0.05, **p < 0.01, ***p < 0.001, ****p < 0.0001.

To validate findings of CCL26 from RNAseq analyses, we performed quantitative RT-PCR and protein analyses of CCL26 following IL-13 stimulation and AHR activation (Figure 6). Analysis of the canonical AHR target gene *CYP1A1* as a positive control in HaCaT cells demonstrated that treatment with the AHR agonists FICZ and tapinarof induced *CYP1A1* expression, which was variably blunted by the AHR antagonist GNF351 (Figures 6A,B). When HaCaT cells were treated with IL-13, *CCL26* was induced significantly (fold change ranges from 86.4 to 1,600). The induction of *CCL26* expression by IL-13 was significantly blunted by both FICZ (mean 86.4 fold with IL-13 alone vs. mean 18.2 fold with IL-13 + FICZ, *p* < 0.001, Figure 6C) and tapinarof (mean 1,600 fold with IL-13 alone vs. mean 550 fold with IL-13 + Tapinarof, *p* < 0.0001, Figure 6D). Protein levels of CCL26 measured by ELISA under these same conditions verified the significant blunting of CCL26 protein levels by FICZ from a mean of 813 pg/ml after IL-13 stimulation to 624 pg/ml after IL-13 and FICZ stimulation (*p* = 0.029, Figure 6E) and mean of 878 pg/ml following IL-13 stimulation to 663 pg/ml following IL-13 and tapinarof stimulation (*p* < 0.0001, Figure 6F), though the absolute amount of protein was only decreased by approximately 20%. These results validate that HaCaT cells are responsive to AHR and IL-13 and that IL-13–dependent CCL26 expression and protein production are both attenuated by AHR activation.

### AHR activation does not alter total STAT6, pSTAT6, or STAT6 nuclear translocation

We hypothesized that AHR mediates its effect on the IL-13 response by regulating the activity of STAT6 [a downstream transcription factor activated by IL-13 and IL-4, which regulates *CCL26* expression (52)]. To confirm that AHR is activated by FICZ, we analyzed AHR cellular localization after 24 h of FICZ treatment, IL-13 treatment, or both FICZ + IL-13 treatment. AHR was mainly present in the cytosol in both control (1% DMSO) and IL-13–treated HaCaT cells, whereas the cytoplasmic fraction was significantly decreased (and nuclear fraction increased) in the FICZ-treated and FICZ + IL-13–treated HaCaT cells (Supplementary Figure S3A). Densitometry of nuclear and cytoplasmic fractions of AHR (Supplementary Figure S3B) showed that FICZ treatment dramatically increased nuclear translocation of AHR and that IL-13 did not affect this translocation. These data confirm that FICZ activates nuclear translocation of AHR and that IL-13 does not affect this process.

**Figure 7:**
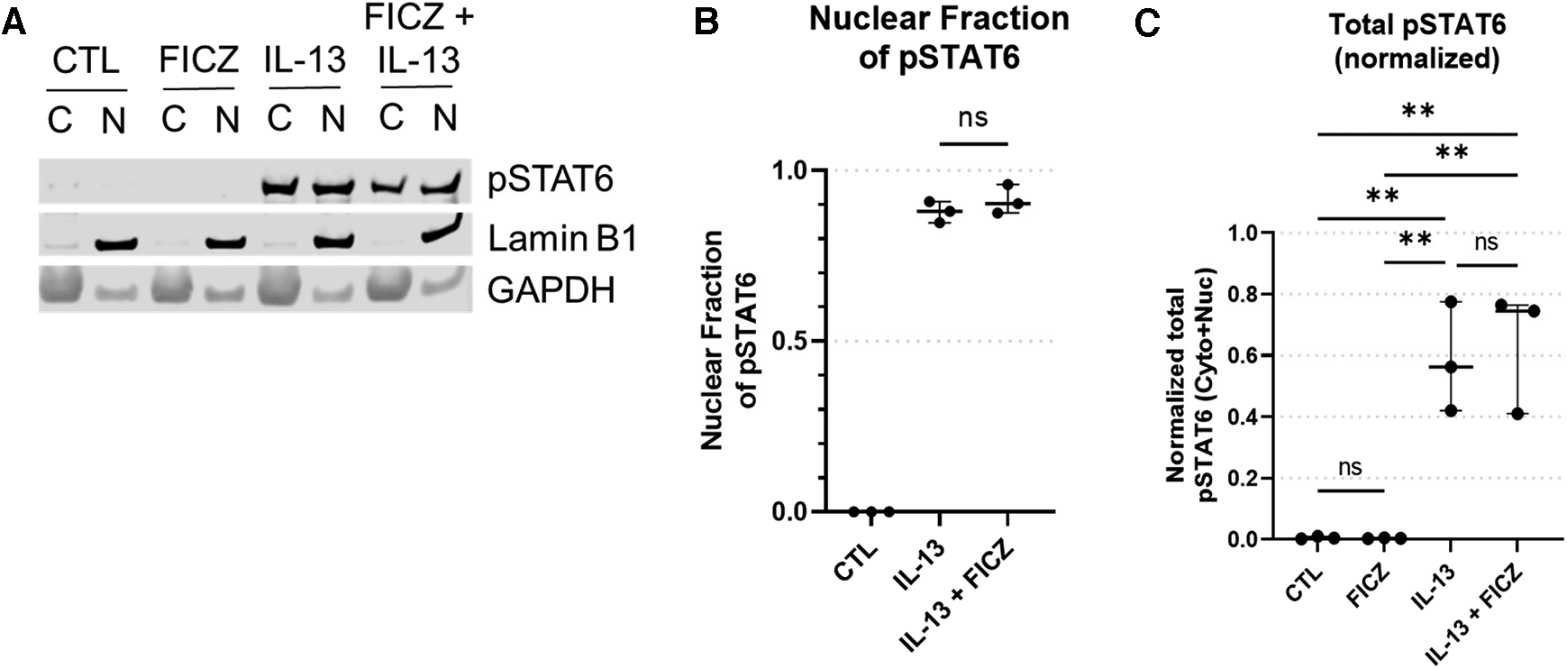
Western blot of pSTAT6 in HaCaT cells. All treatment reagents were added simultaneously without pre-incubation of any reagents for 1 h prior to extraction. (**A**) Representative pSTAT6 western blot using cytoplasmic and nuclear fractions of the same sample after 1 h of treatment, with nuclear marker LaminB1 and cytoplasmic marker GAPDH. Control (CTL) is untreated, IL-13 is 10 ng/ml, with “*C*” designating cytoplasmic and “*N*” designating nuclear fractions of the same sample, respectively. Intensity of bands from (**A**) were quantified [Median +/− interquartile range shown (*n* = 3 replicates per group)], and the normalized nuclear pSTAT6 fraction is shown in (**B**) [nuclear pSTAT6/(nuclear pSTAT6 + cytoplasmic pSTAT6)]. Specifically cytoplasmic pSTAT6 was normalized to cytoplasmic GAPDH and nuclear pSTAT6 was normalized to nuclear LaminB1. Total pSTAT6 (normalized nuclear pSTAT6 intensity + normalized cytoplasmic pSTAT6 intensity) is shown in (**C**). One-Way ANOVA with Tukey's test for multiple comparisons; ns, not significant, **p < 0.01.

To determine whether AHR activation impacts STAT6 phosphorylation or nuclear translocation, we performed western blots for pSTAT6 (Figure 7). pSTAT6 was not present in untreated or FICZ-treated samples. As a positive control, pSTAT6 was present in both cytosolic and nuclear fractions of the IL-13 treatment group and the IL-13 + FICZ treatment group (Figure 7A). Quantification of pSTAT6 showed that the nuclear fraction of pSTAT6 was comparable between IL-13– and IL-13 + FICZ–treated cells, indicating that AHR activation did not alter nuclear translocation of pSTAT6 (Figure 7B). Of note, when total pSTAT6 was quantified (nuclear pSTAT6 + cytoplasmic pSTAT6, shown in Figure 7C), there was no significant difference in FICZ-treated cells, showing that AHR activation did not affect STAT6 activation. A significant increase in total pSTAT6 with IL-13 and IL-13 + FICZ groups was observed (*p* < 0.01 when any IL-13–treated group was compared to either control or FICZ-treated cells), though there were no significant differences noted between IL-13 and IL-13 + FICZ groups (Figure 7C).

To confirm the impact of AHR activation or IL-13 treatment on total STAT6 levels, quantitation of total STAT6 revealed that only IL-13 treatment caused an increase in total STAT6 levels and that AHR activation with FICZ did not affect total STAT6 (Supplementary Figure S4). Together, these data show that in our HaCaT model system, FICZ activates AHR nuclear translocation, which is not affected by IL-13; additionally, AHR activation by FICZ does not change total pSTAT6, nuclear translocation of pSTAT6, nor total STAT6 levels, which suggest that attenuation of IL-13 signaling by activation of AHR must be occurring independent of intracellular STAT6 expression, activation or trafficking. This finding is highly suggestive that alterations in gene expression between AHR and STAT6 activation are mediated by changes in the nucleus, which could include DNA binding competition.

### Enrichment of AHR binding motifs in active chromatin regions of DEGs from keratinocytes of patients with AD

**Figure 8:**
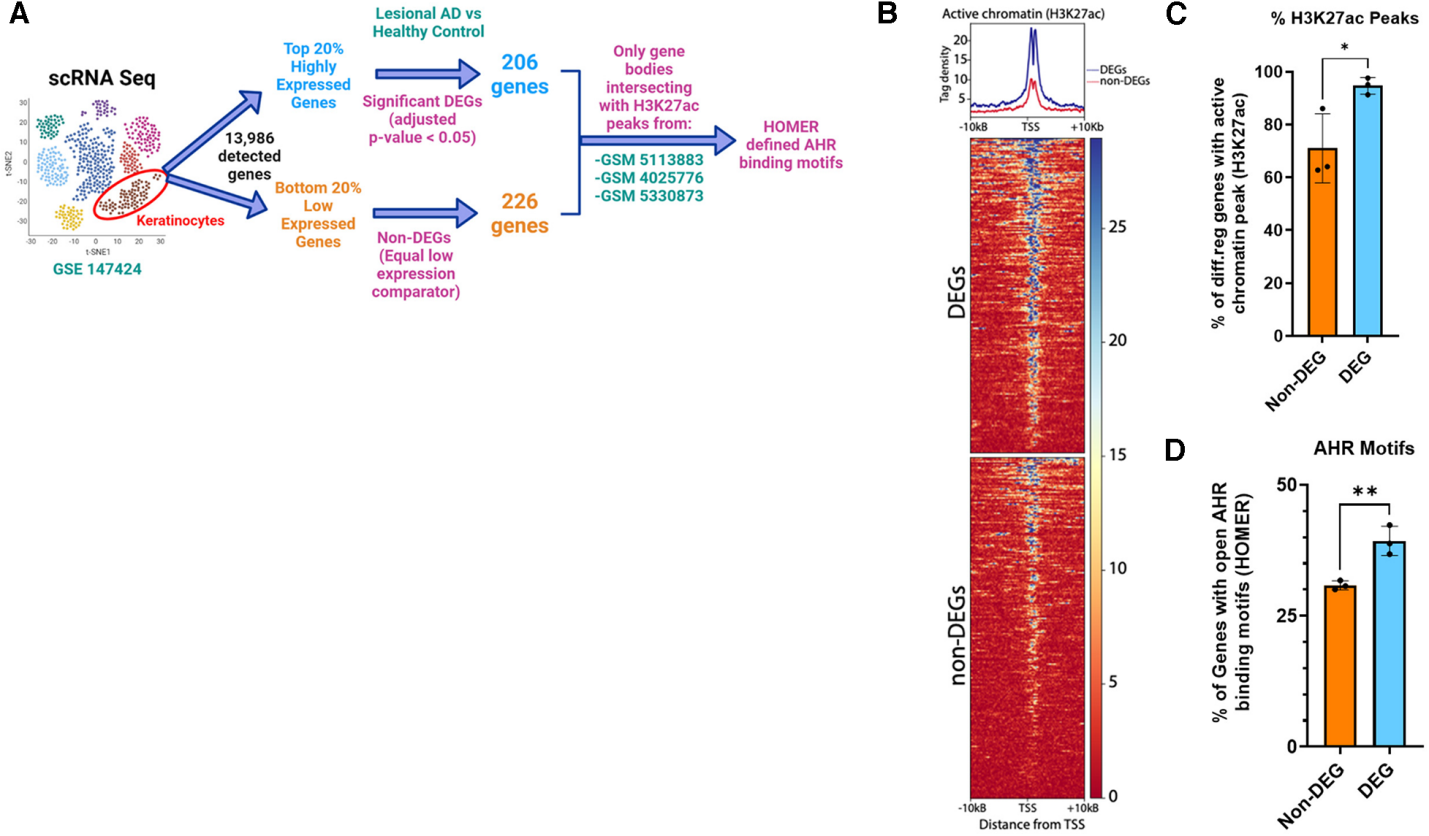
Enrichment of AHR binding motifs in open chromatin regions (H3K27ac) of differentially expressed genes from keratinocytes of patients with atopic dermatitis (AD) vs. healthy controls. (**A)** Flow diagram of analysis. Keratinocytes from lesional AD vs. healthy controls were identified from scRNAseq data (GSE147424, hypothetical tSNE plot shown). Next, gene lists for the top 20% and bottom 20% expressed genes (−2,800 each) were searched for any DEGs (adjusted *p* < 0.05) from the lesional AD vs. healthy control comparison. We found that 206 genes were differentially expressed in the highly expressed gene group (DEGs), and that no DEGs were differentially expressed among the low expressed gene group (non-DEGs). We chose 226 genes, all of which had the exact same lowest detected level of expression, to be the non-DEG (equal low expression comparator) group. (**B)** H3K27ac tag density (using H3K27ac ChIP-seq peaks from 3 different human keratinocyte data sets: GSM5113883, GSM4025776, and GSM5330873) were compared in DEGs (206) vs. non-DEGs (226). (**C**) Percentage of DEGs that intersected with an active chromatin peak of DEGs vs. non-DEGs. (**D**) Percentage of genes with AHR binding motifs between DEGs vs. non-DEGs. Unpaired *t*-test (*n* = 3); *p < 0.05, **p < 0.01. Flow diagram in (**A**) created with BioRender.com.

Because AHR activation did not affect pSTAT6 nuclear translocation and given the overlap of DEGs between IL-13 and AHR, we hypothesized that STAT6 competes with AHR on DNA binding as a potential mechanism of AHR-induced changes to IL-13–regulated genes. We sought to determine whether AHR binding motifs were enriched among DEGs from lesional keratinocytes (compared to healthy controls). Utilizing available scRNAseq data from keratinocytes of patients with AD (lesional sample) compared to healthy controls (from GSE147424), we identified the top 20% highly expressed genes based on absolute fold change (approximately 2,800 genes). From this top 20%, we found that 206 genes were DEGs (adjusted *p* < 0.05 when comparing Lesional AD keratinocytes with healthy control keratinocytes). There were no DEGs among the lowest 20% expressed genes. In order to find a group of genes that would serve as an equivalent low expression comparator group, we found that the lowest expressed 226 genes all had the exact same level of absolute expression, and this group was selected and named “Non-DEGs”. Of these genes, we analyzed their likelihood to have active chromatin by intersecting with ChIP-seq data from human keratinocytes (GSM5113883, GSM4025776, and GSM5330873). BedTools intersect was used to determine whether any part of the gene body overlapped with areas of active chromatin as determined by H3K27 acetylation (H3K27ac) (Figure 8). As expected, tag density of H3K27ac-marked active regions of chromatin were centered on the transcriptional start site (TSS) of each gene (Figure 8B). We found that significantly more AD DEGs overlapped with H3K27ac-enriched active chromatin regions than did Non-DEGs (94.7% vs. 71.0%, *p* = 0.038) (Figure 8C). Finally, H3K27ac-enriched gene regions were analyzed for consensus AHR binding motifs. Highly DEGs contained a significantly higher proportion of AHR binding motifs than did Non-DEGs (39.3% vs. 30.9%, *p* = 0.007) (Figure 8D). Collectively, these data suggest that AHR signaling may attenuate IL-13–induced responses in keratinocytes by competing with pSTAT6 on DNA binding.

## Discussion

In this study, we presented a collective line of evidence demonstrating that AHR signaling is altered in keratinocytes of patients with AD compared to controls. We further demonstrated that activation of AHR signaling by delivery of AHR ligands induces epithelial barrier genes and attenuates IL-13–mediated induction of key AD signature genes, including *CCL26*. Aiming to elucidate the mechanism by which AHR attenuates the IL-13 response, we hypothesized that AHR interacts with and restrains STAT6. However, we demonstrated that AHR does not interfere with total STAT6 protein expression, STAT6 phosphorylation, nor nuclear translocation.

These collective data raise the question of “Why would AHR target genes display decreased expression in AD?”. The first plausible explanation, supported by our new finding that AHRR expression is increased in AD (Figure 1), suggests that AHRR may act on lesional keratinocytes to prevent AHR target gene expression. A second plausible explanation may be potential insufficiency of protective AHR ligands in the AD patient environment (possibly due to imbalance of microbial-produced ligands or dietary molecules), leading to the retention of AHR outside of the nucleus. These external environmental effects were not measured by our model system and should be monitored on patients' samples. The third explanation could be attributed to the competition between STAT6 and AHR on binding of shared target genes. Given that STAT6 is activated by allergic stimuli such as IL-13 and IL-4, and these cytokines are elevated in AD patients compared to control patients, STAT6 is already enriched in the nuclei of keratinocytes of AD patients. Upon AHR nuclear translocation, AHR encounters nuclear STAT6, and competes with STAT6 on DNA binding of shared genes, resulting in decreased transcription of AHR target genes. In contrast, keratinocytes of healthy individuals lack nuclear STAT6. Consequently, upon AHR nuclear translocation, AHR can freely bind and regulate its target genes.

**Figure 9:**
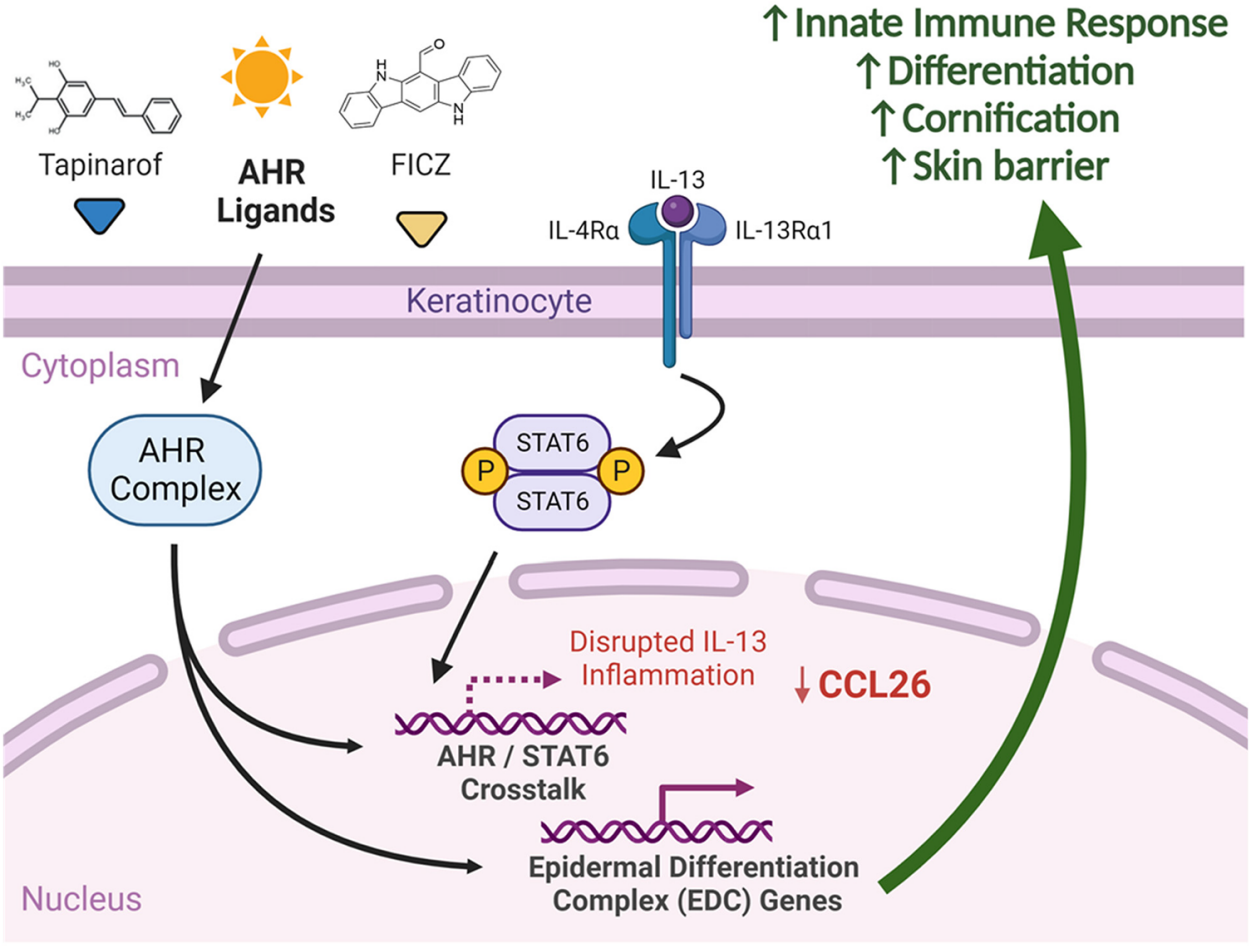
Summary of AHR and IL-13 signaling in keratinocytes. We suggest that significant crosstalk exists between AHR and STAT6 affecting expression of shared genes. Delivery of select AHR ligands, such as FICZ and tapinarof, to keratinocytes has the potential to attenuate IL-13-mediated STAT6 responses, including CCL26 expression. AHR activation with select ligands induces differentiation and skin barrier genes, which may explain in part the efficacy of AHR agonists in atopic dermatitis. Created with BioRender.com.

It is important to note that AHR forms complexes with various transcription factors that have different target genes based on ligands and the environmental context. Shared co-factors may be required for transcriptional activity of both AHR and STAT6 and could be a basis for this competition. The presence of STAT6 may interfere with AHR transcriptional activity, contributing to the observed decrease in the expression of AHR target genes in the context of AD (53) (summarized in Figure 9).

Prior to scRNAseq, bulk RNAseq and immunohistochemistry were the best techniques available to locally probe approximate gene expression in keratinocytes of subjects with AD. AHR expression has generally been shown to be increased in the epidermis of patients with AD compared to controls (54–56) . A recent study of bulk RNAseq of biopsies from patients with AD at different ages by Renert–Yuval et al. showed decreased AHR expression in normal healthy skin compared with lesional and non-lesional skin, though this was only in children 0–5 years old (*n* = 17) (57). Since the data that we used in Figure 1 was obtained from adult patients, further study of age-associated AHR expression would be required to reconcile why AHR expression in the skin of adults with AD is variable. *CYP1A1* expression, in contrast, has shown variable levels of expression in skin of patients with AD. Hidaka et al. showed variable levels of expression trending toward an increase in epidermal cells (58). Kim et al. also showed increase of CYP1A1 expression in AD compared to controls by bulk PCR of skin biopsy samples (54). However, Hong et al. showed low levels of CYP1A1 expression with fluorescent immunohistochemistry, in agreement with our results (55). One possible cause for these discrepancies is the bulk nature of the specimens, highlighting the importance of the single-cell resolution of gene expression afforded by newer technologies, such as scRNAseq. Our analysis of keratinocyte-specific gene expression from available scRNAseq data confirms the general trend seen in immunohistochemistry and bulk RNAseq, wherein AHR expression is increased in lesional skin without consistent activation of canonical AHR target genes, which may be mediated by AHRR and other genes. While further work is needed, our data suggests that dysregulation of the AHR pathway in keratinocytes may contribute to the pathogenesis of AD.

Keratinocytes are uniquely positioned to have exposure to AHR ligands and precursors of AHR ligands. First, the skin is exposed to UV radiation, which is required for FICZ generation from endogenous tryptophan (59). Another source of AHR ligands for keratinocytes are commensal microbes, which produce a variety of indoles and related AHR ligands in the normal course of their metabolism (4). Notably, subjects with AD have altered microbiota (typically switching from *Staphylococcus epidermitis* dominant to *Staphylococcus aureus* dominant). This calls for more detailed studies of AHR ligand production by these commensal bacteria. Further, impaired barrier function of AD keratinocytes can alter exposure to environmental AHR ligands, both from commensal bacteria and pollutants (namely particulate matter and products of combustion). Given the importance of environmental factors on AD risk and the ability for AHR to interact with many environmental ligands, it is plausible that AD pathogenesis relies on variable exposures to AHR ligands and precursor molecules (some protective, some pathogenic). Further study is needed to put AHR ligand exposures into context, though it is intriguing to consider AHR as a key piece of the environmental puzzle of AD and the rising incidence of other allergic diseases (60).

Our study is the first to directly compare lesional DEGs from AD keratinocytes to AHR activation of an *in vitro* keratinocyte cell model (Figure 3). The shared AD and AHR target genes mostly reversed direction in expression between AD and AHR activation states and were involved with keratinocyte development and skin barrier function, which support a possible mechanism by which AHR activation might improve AD. Other studies to date support this relationship between AHR activation and improved skin barrier function. For instance, Van den Bogaard et al. used skin organoids derived from primary keratinocytes of patients with AD and showed that coal tar acted through AHR to restore *FLG* expression and other markers of keratinocyte differentiation (6). Furthermore, Tsuji et al. showed that in primary human keratinocytes, AHR activation with FICZ and Glyteer (soybean tar) could induce the expression of the barrier gene *FLG* and that FICZ and Glyteer can restore the *FLG* expression that was reduced by IL-4 treatment, and that AHR activation mediated these effects via OVOL1 (7). Notably, filaggrin (FLG) is a key protein involved in the skin barrier, and mutations in *FLG* are associated with AD (61). Tapinarof has been shown by Smith et al. to induce expression of epidermal differentiation genes in keratinocytes and to improve inflammation in an AHR-dependent manner using both a human *ex vivo* air-liquid interface culture model and an imiquimod dermatitis mouse model (8).

Notably, the crosstalk between AHR and IL-13 does not appear to be limited to just skin barrier function; we compared *in vitro* AD-like conditions with and without AHR activation and demonstrated that the shared DEGs included not only skin barrier function (extracellular matrix, external structure), but also inflammatory processes, such as chemotaxis, humoral response, alternative complement, and IL-17 signaling (Figure 4C). Other studies to date have also shown the ability of AHR to regulate type 2 inflammation in epithelial cells. AHR has been shown to bind to the TSLP promoter to downregulate its expression in mouse keratinocytes, and though TSLP was not among the several DEGs found in our study, the overall mechanism would be consistent with our findings for CCL26 (62). Another study revealed relevance of AHR signaling in proton pump inhibitor (PPI) responsiveness of esophageal epithelial (EPC2) cells and reversal of approximately 20% of the IL-13 transcriptome, consistent with some of the expression reversals that we describe here (63). In another study, coal tar activation of AHR prevented IL-4– and IL-13–dependent CCL26 expression in human primary keratinocytes derived from patients with AD, consistent with our findings (6). Of note, they showed that pSTAT6 was decreased after addition of coal tar to skin organoids pre-treated with both IL-4 and IL-13. This finding contrasts with our results, in which no changes to pSTAT6 were noted, though there were also differences between AHR agonist (FICZ vs. coal tar) and Th2 stimulation (IL-4 and IL-13 vs. IL-13 alone). Other studies of AHR implicate AHR- and NRF2-dependent dephosphorylation of STAT6 as an anti-inflammatory mechanism in HaCaT cells (64). While we did not observe changes in STAT6 phosphorylation with AHR activation (Figure 7), our analysis of the 100 genes affected by IL-13 and subsequently reversed by FICZ identified enrichment of genes associated with the NRF2 pathway (Supplementary Figure S2C). The NRF2 pathway is significantly intertwined with that of AHR (65), and further studies must reconcile both as we resolve the dynamic relationship between AHR and inflammatory skin disease. Our work here adds to the mounting evidence that crosstalk in AHR and STAT6 is likely to play a role in AD.

It is important to recognize that AHR activation is not exclusively beneficial to skin, as studies utilizing the AHR ligands TCDD and PCBs (in addition to case studies of human exposures) demonstrate intense cystic dermatitis and inflammation (termed “chloracne” in humans) (66). In mice, a constitutively active AHR mutant leads to a dermatitis phenotype (67). One factor likely contributing to this differential response to AHR activation is the metabolism and stability of the many known AHR ligands; for example, TCDD has a half-life between 5 and 10 years in the human body (68) compared to FICZ's half-life of a few hours (69, 70). Another factor complicating the study of AHR activation in the skin is the role of AHR signaling on immunologically active cells. The importance of AHR to T helper 17 (Th17) cells is well known, and its effects on innate lymphoid cells, dendritic cells, macrophages, and others cannot be discounted (71). However, we suggest that the role of AHR in keratinocytes by natural ligands like FICZ is particularly important in AD. Unraveling the nuances of AHR activation as a therapeutic target in AD will require more detailed mechanistic studies controlling for the many AHR ligands, cell types, and even AD endotypes (72).

Our study is limited by the small number of subjects used in some of the RNAseq studies of keratinocytes; however, these data will evolve as data from larger studies are published. Additionally, use of keratinocytes from biopsies of subjects with AD treated with and without AHR ligands, such as tapinarof, would provide much more direct comparisons of the effects of AHR activation in AD than *in vitro* models. We acknowledge that more complicated models, such as air-liquid interface culture or skin organoids, may prove to be more representative of human skin, though future studies can explore these further. Elucidation of the roles of NRF2 and OVOL1, both transcription factors that have known associations with AHR in keratinocytes, should also be explored further in our model system. Finally, more in-depth studies of DNA binding competition between AHR and pSTAT6 are needed to understand crosstalk between these two transcription factors in AD.

In conclusion, we provide evidence that AHR signaling is disrupted in keratinocytes of subjects with AD and that an overlap exists between the genes that are altered by IL-13 and genes that are regulated by AHR activation. These overlapped genes, including CCL26, are enriched for skin barrier functions and innate immune responses. AHR activation does not appear to alter STAT6 levels, activation, nor nuclear translocation, and we provide evidence that DNA binding may be the primary means by which AHR activation alters IL-13 signaling. Further study of AHR in AD will help clarify to what extent this environmental sensor can regulate responses to allergic disease.

## Data Availability

The datasets presented in this study can be found in online repositories. The names of the repository/repositories and accession number(s) can be found in the article/**Supplementary Material**.
