# Supplementary material for: Aryl hydrocarbon receptor and IL-13 signaling crosstalk in human keratinocytes and atopic dermatitis
Source: Front Allergy. 2024 Jan 26;5:1323405. doi: 10.3389/falgy.2024.1323405 (PMC10853333; doi:10.3389/falgy.2024.1323405)
Supplement: Supplementary file 2 [file Datasheet1.docx]

**Supplemental Figure 1**


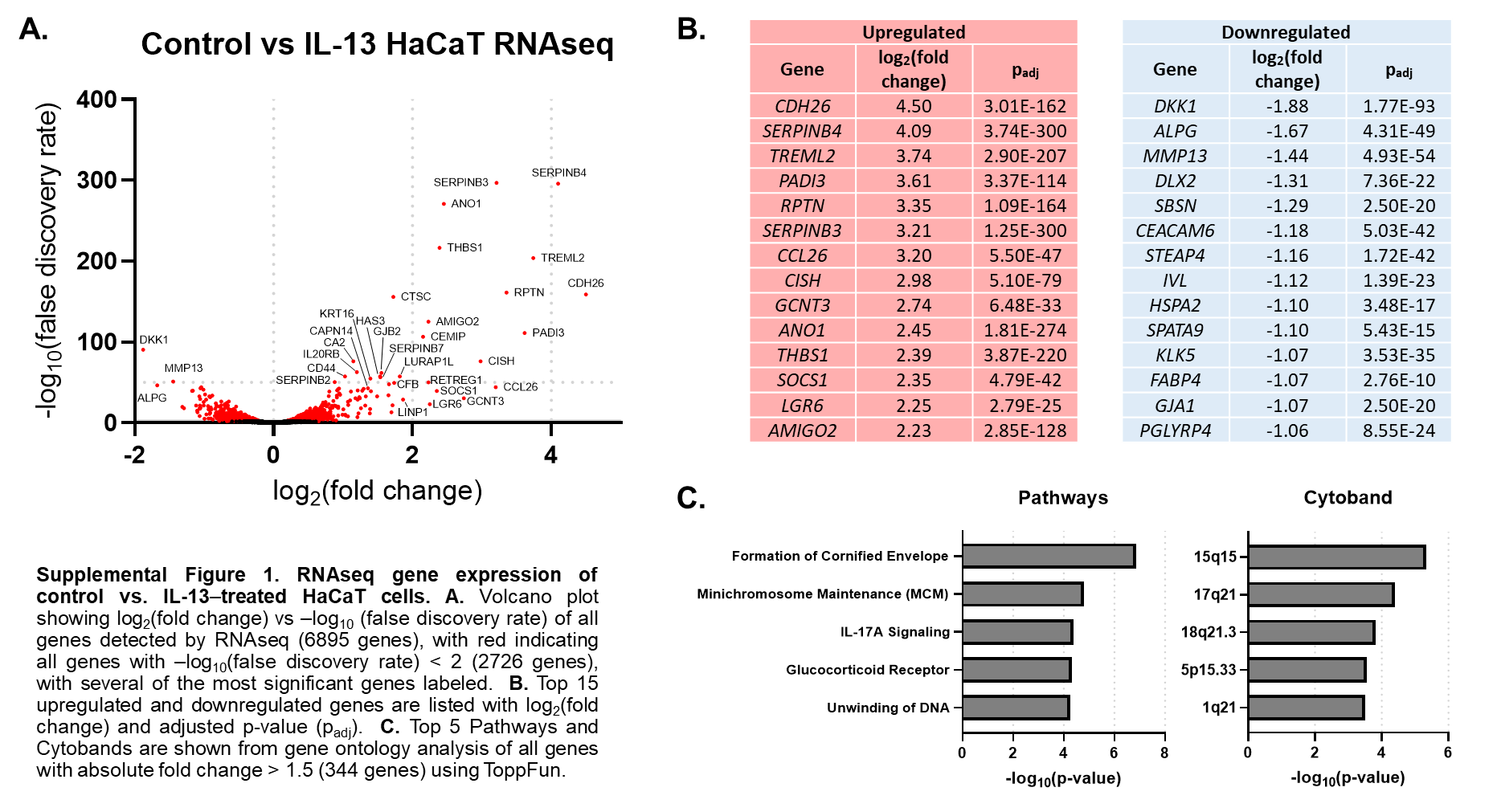


**Supplemental Figure 2**


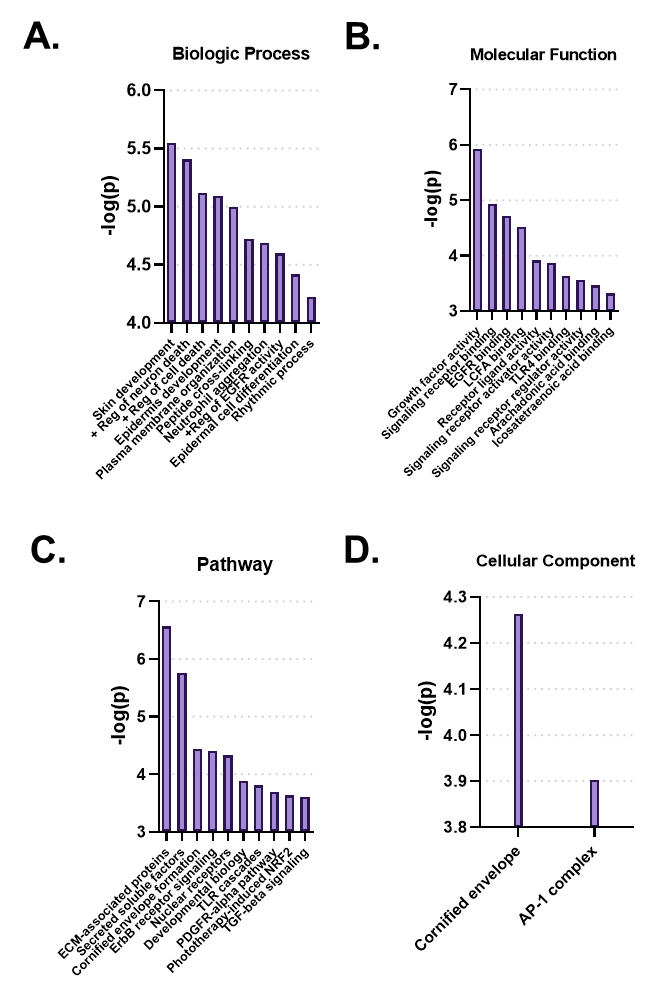


**Supplemental Figure 2. Gene Ontology Analysis of 100 Genes Changed by IL-13 and reversed by AHR activation.** Genes that showed reversal of expression pattern from IL-13 to IL-13+FICZ (Supplemental Figure 1) were analyzed. Shown are the top 10 biologic processes (**A**), the top 10 molecular functions (**B**), the top 10 pathways (**C**), and the top 2 cellular components (**D**). All values are expressed as -log(p-value). + Reg, upregulation; EGFR, epidermal growth factor receptor; LCFA, long-chain fatty acid; TLR, toll-like receptor; ECM, extracellular matrix; ErbB, epidermal growth factor receptor family; PDGFR, platelet-derived growth factor receptor; TGF, transforming growth factor; AP, activating protein.

**Supplemental Figure 3**


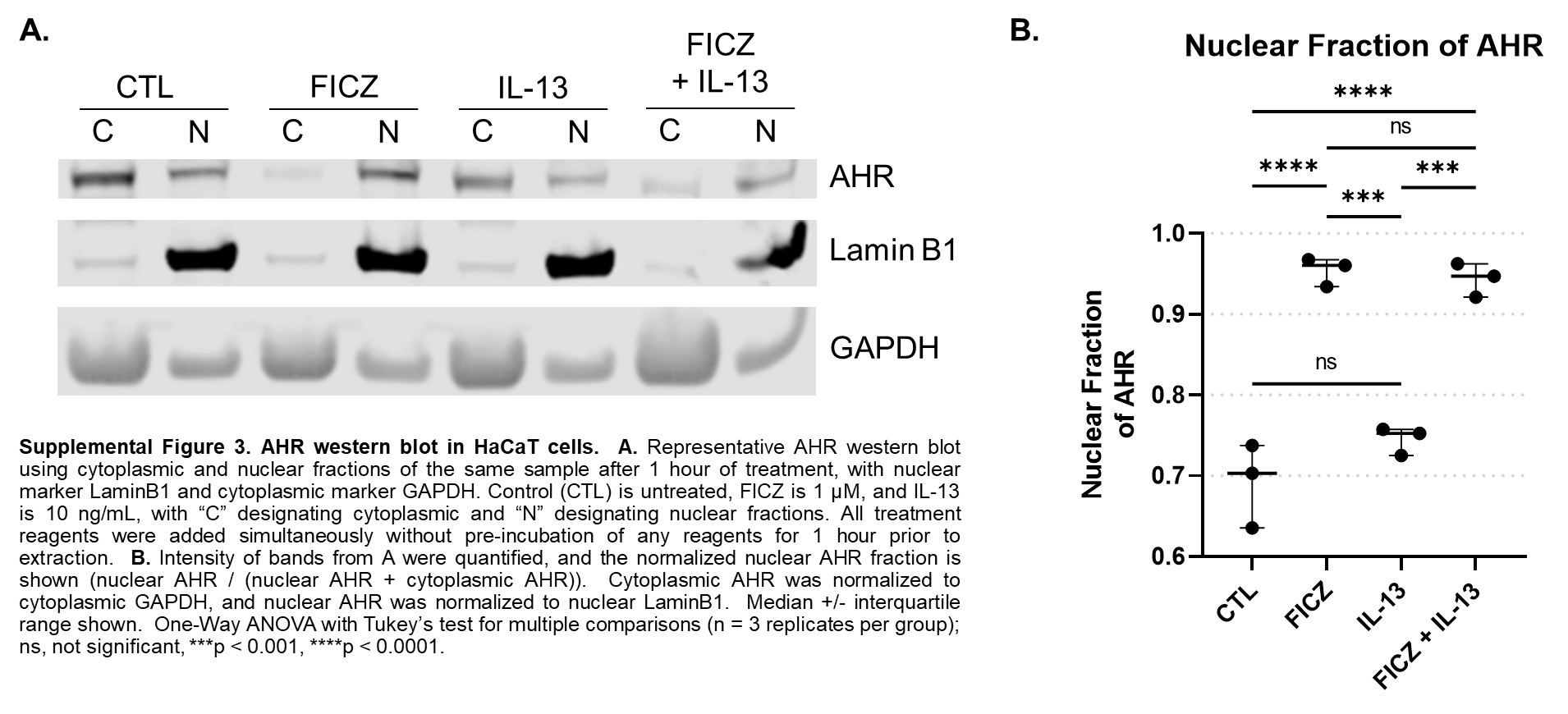


**Supplemental Figure 4**

**
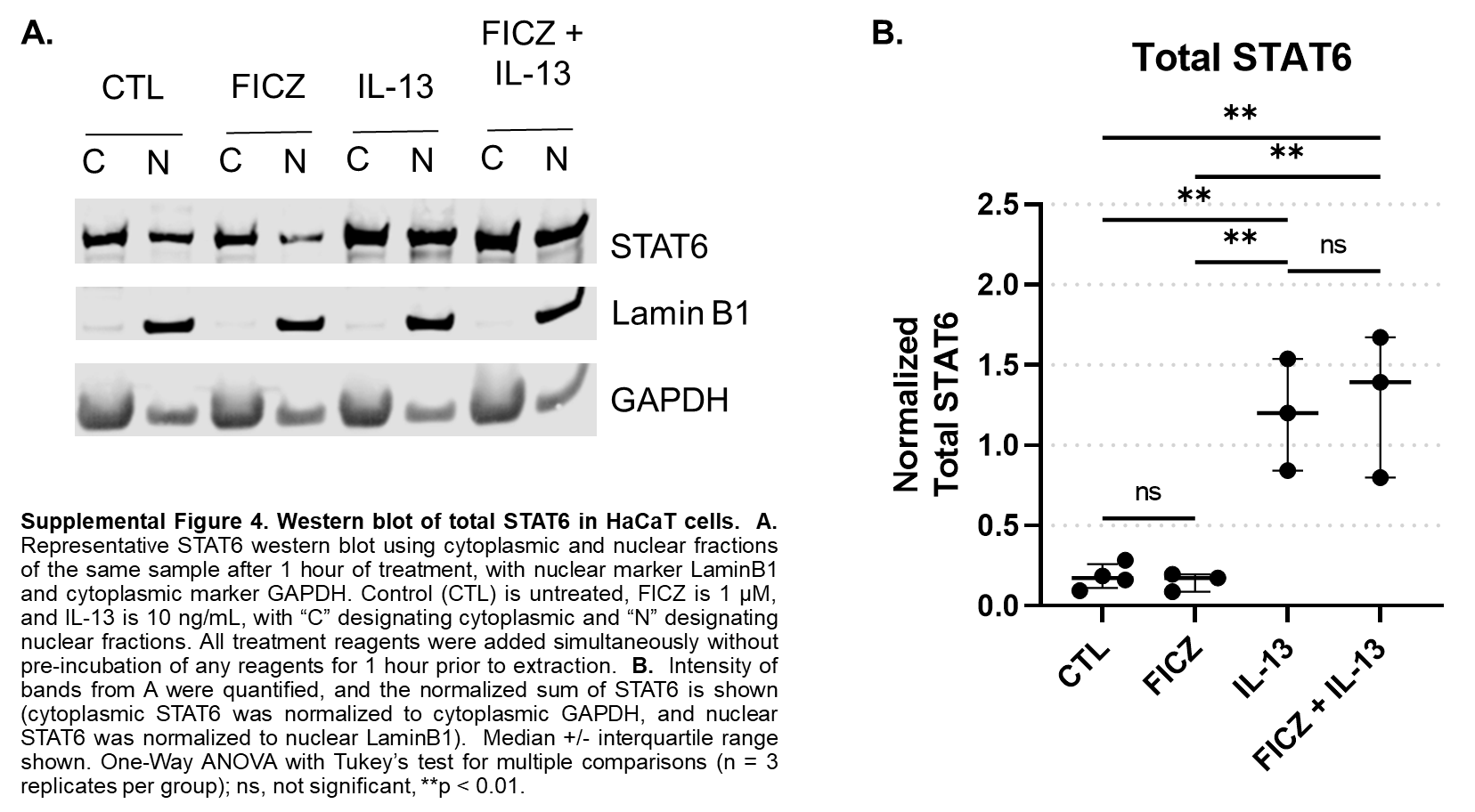
**
